# Mitochondrial Genome of *Phlebia radiata* Is the Second Largest (156 kbp) among Fungi and Features Signs of Genome Flexibility and Recent Recombination Events

**DOI:** 10.1371/journal.pone.0097141

**Published:** 2014-05-13

**Authors:** Heikki Salavirta, Ilona Oksanen, Jaana Kuuskeri, Miia Mäkelä, Pia Laine, Lars Paulin, Taina Lundell

**Affiliations:** 1 Microbiology and Biotechnology, Department of Food and Environmental Sciences, University of Helsinki, Helsinki, Finland; 2 Institute of Biotechnology, DNA Sequencing and Genomics Laboratory, University of Helsinki, Helsinki, Finland; University of Wisconsin - Madison, United States of America

## Abstract

Mitochondria are eukaryotic organelles supporting individual life-style *via* generation of proton motive force and cellular energy, and indispensable metabolic pathways. As part of genome sequencing of the white rot Basidiomycota species *Phlebia radiata*, we first assembled its mitochondrial genome (mtDNA). So far, the 156 348 bp mtDNA is the second largest described for fungi, and of considerable size among eukaryotes. The *P. radiata* mtDNA assembled as single circular dsDNA molecule containing genes for the large and small ribosomal RNAs, 28 transfer RNAs, and over 100 open reading frames encoding the 14 fungal conserved protein subunits of the mitochondrial complexes I, III, IV, and V. Two genes (*atp6* and tRNA-Ile^GAU^) were duplicated within 6.1 kbp inverted region, which is a unique feature of the genome. The large mtDNA size, however, is explained by the dominance of intronic and intergenic regions (sum 80% of mtDNA sequence). The intergenic DNA stretches harness short (≤200 nt) repetitive, dispersed and overlapping sequence elements in abundance. Long self-splicing introns of types I and II interrupt eleven of the conserved genes (*cox1,2,3*; *cob; nad1,2,4,4L,5; rnl; rns*). The introns embrace a total of 57 homing endonucleases with LAGLIDADGD and GYI-YIG core motifs, which makes *P. radiata* mtDNA to one of the largest known reservoirs of intron-homing endonucleases. The inverted duplication, intergenic stretches, and intronic features are indications of dynamics and genetic flexibility of the mtDNA, not fully recognized to this extent in fungal mitochondrial genomes previously, thus giving new insights for the evolution of organelle genomes in eukaryotes.

## Introduction

*Phlebia radiata* Fr. is a saprobic, wood-colonizing and white-rot type of wood decay causing polypore fungal species of the class Agaricomycetes, phylum Basidiomycota, and is encountered in Eurasian and North-American forests generally on dead angiosperm wood [1], [2]. We initiated *de novo* whole genome sequencing of *P. radiata* due to its notable biotechnological abilities in decomposition of wood components and lignocelluloses, and in oxidation and conversion of synthetic and milled wood lignin, and lignin model compounds [3]–[5]. The fungus is also efficient in degradation of xenobiotics and production of lignin-converting oxidoreductases like lignin peroxidases and manganese peroxidases, and laccase [3]–[6].

The draft assembly of 454-sequenced *P. radiata* genome resulted first with ca. 300x coverage of a single scaffold and circular dsDNA molecule of over 156 kbp in size, which turned out to be the mitochondrial genome. Mitochondria are cellular organelles of eukaryotes which support individual life-style and generate proton motive force for production of ATP and energy *via* respiration [7]–[9]. Mitochondria are also known to participate in many other indispensable cellular processes such as calcium homeostasis, cell aging and apoptosis, iron metabolism, and synthesis of iron-sulphur clusters for oxidoreductive enzymes [9]–[12].

Essence of mitochondria is accepted to arise from endosymbiosis [13], [14], most reliably of the SAR11 clade ancestor marine bacterium (pelagibacteria) [15]. Adaptation to the host organism has resulted with co-evolution of the mitochondrial genome and gene flow to the host genome [7], [8], [16], [17]. It was previously considered that mitochondrial genomes are small and compact, according to information mostly achieved from metazoa, such as the only 16 kbp-size human mitochondrial genome [9]. This notion has, together with the retarded mtDNA sizes, previously led to the proposal of the “vanishing mitochondria”, especially in fungi [8].

Complete genome sequencing on eukaryotic micro- and macro-organisms has, however, demonstrated a higher degree of mitochondrial genome structural complexity, and variation in the mtDNA size than was previously realized. Complicated network of mini-circle mtDNAs are present in the basal body mitochondrion of the Kinetoplastida protozoa [18], when the largest mt genomes are described for Embryophyta and Charophyta [19], [20], i.e. for land plants and green algae. In angiosperm flowering plants, the mtDNA varies highly in size (200 kbp to 11 Mbp) and may be organized to multiple chromosomes [20]–[22]. So far, the largest plant mt genomes were recently sequenced for *Silene* species as complex entities with up to 128 circular-mapping chromosomes [22].

Currently, 162 fully sequenced and annotated fungal mtDNA sequences are publicly available. The overwhelming majority (124) of these belong to Ascomycota [19]. Basidiomycota are the second best represented fungal phylum with 21 complete mt genomes [19], [23]–[28]. The other publicly available fungal mtDNAs include a few genomes from species of Blastocladiomycota, Chytridiomycota, Glomeromycota, Monoblepharidomycota, one Cryptomycota (*Rozella allomycis*), and three previous Zygomycota, now *incertae sedis* species [19]. Exceptionally, the Microsporidia and the anaerobic fungi of Neocallimastigomycotina lack traditional mitochondria, which were modified to other cellular organelles such as hydrogenosomes [8]. Most fungal mt genomes are characterized as single circular dsDNA molecules [7], [8], [23]–[29], when linear or transiently linear chromosome organization was reported for a few species [7], [8], [30]–[32].

Fungal mtDNA generally encloses 14 essential protein-coding genes (*atp6*,*8*,*9*; *cob*, *cox1*-*3*, *nad1*-*6*, and *nad4L*) for protein subunits of the mitochondrial complexes I, III, IV, and V required for electron transfer and oxidative phosphorylation. Another common, but more randomly distributed fungal mtDNA-contained gene is *rps3*, which encodes the small ribosomal subunit protein S3. Other typical genes to fungal mtDNA are the small (*rns*) and large (*rnl*) subunit mitochondrial rRNAs, and a tRNA set - generally at least 23 unique anticodons - sufficient to translate the mtDNA-encoded proteome [7], [8], [10], [23]–[28]. However, exceptions are not unusual. For example, in the Ascomycota budding yeast *Saccharomyces cerevisiae*, the 85.8 kbp mtDNA includes over 40 genes encoding e.g. 24 tRNAs and the two rRNAs, but lacks two of the 14 conserved protein-coding genes (those for Complex I subunits) [29].

Together with our study, recent genome sequencing reports indicate that fungal mitochondrial genomes have a much higher degree of variation in size, gene content, genomic organization and gene order, and gene intron-exon construction than has been realized previously. We acknowledge that the high number of intron-homing endonucleases (HEs) recognized in the *P. radiata* mtDNA may play an editing role, both in genome replication and gene transcription, as well as an integrating role for intron and gene transposition in the mtDNA. Another unique feature is the duplicated “mirror” region in the genome, which together with the repetitive-element dense sections may promote both DNA recombination and gene transcription. We also discuss mtDNA-encoded proteome phylogeny in relation to tRNA evolution and ORF codon usage, in regard to the currently accepted concept of fungal systematics.

## Materials and Methods

### Fungal Isolate and Cultivation

*Phlebia radiata* Fr. strain 79 (FBCC0043) was originally isolated from a distinguishable fruiting body found in South Finland on white-rot decayed alder (*Alnus incana*), and maintained in the Fungal Biotechnology Culture Collection at the Department of Food and Environmental Sciences, University of Helsinki, as living mycelium on 2% (wt/vol) malt extract, 1.5% (wt/vol) agar slants under paraffin oil at 12°C. Species identification is based on both macroscopic features of the original fruiting body and mycelium, as well as at molecular level on ribosomal 18S rRNA gene and ITS1-5.8S-ITS2 bar coding sequences [33]. For isolation of total DNA, the fungus was cultivated in liquid 2% (wt/vol) malt extract broth for 10 days at 28°C in the dark. After cultivation, the mycelial mats were harvested and washed with cold ultrapure water, frozen to −20°C, and lyophilized.

### DNA Isolation

Dry mycelium was quickly ground in acid-washed and autoclaved mortar. DNA was isolated using a modified version of the hot-CTAB extraction at 65°C [34], followed by phenol-chloroform and 3x chloroform-isoamyl alcohol extractions, and incubation with 0.1 mg/ml Proteinase K (Fermentas) for 30 min at 55°C. Total DNA was precipitated overnight with isopropanol at 4°C, centrifuged at 6500 g 30 min at 4°C, washed twice with 70% ethanol, and subjected to 50 U/ml of RNAseA (Fermentas) treatment at 37°C overnight. After chloroform-isoamyl alcohol extraction, and re-precipitation with ice-cold 94% ethanol overnight at −20°C, DNA was dissolved in sterile TE (10 mM Tris-HCl buffer with 1 mM EDTA, pH 7.5) solution. Integrity an d amount of the isolated total DNA was examined by 1.5% (wt/vol) agarose gel electrophoresis, and using the NanoDrop 1000 Spectrophotometer (Thermo Scientific).

### 454 Sequencing and mt Genome Assembly

Single-stranded template DNA (sstDNA) was sequenced using the 454 sequencing technology with GS FLX Titanium chemistry (Roche, 454 Life Sciences). Number of obtained reads was 1 876 081 containing 752 Mbp of both genomic DNA (gDNA) and mitochondrial DNA (mtDNA). All reads were assembled using Newbler (Roche, 454 Life Sciences) software. Mitochondrial contigs containing high average sequence coverage (approximately 300x) were placed in proper order, resulting with single scaffold, and a finished mtDNA circular genome was defined being 156 348 bp in length. Circularity and sequence orientation, in particular for the large duplicated region, was verified with genome-walking PCR.

### Gene Annotation and Bioinformatic Analyses

The Mold, Protozoan, and Coelenterate Mitochondrial Code and the Mycoplasma/Spiroplasma Code (NCBI translation table 4) was at first assumed for ORF detection. Protein-coding and rRNA genes were annotated by blastp and blastn queries against non-redundant NCBI databases [35]–[37], and localised and annotated in the mtDNA sequence using Artemis [38] software. Intron-exon boundaries of the conserved genes were adjusted manually on the basis of ClustalX [39] multiple Basidiomycota mt coding sequence alignments. Transfer-RNAs were identified with tRNAscan-SE [40]. HEs were recognized by Pfam 26.0 database [41] queries. Protein domain images were generated with ExPASy PROSITE MyDomains Image Creator (http://prosite.expasy.org/mydomains/) and edited in Inkscape version 0.48.2 (http://inkscape.org/). Intron types were determined with RNAweasel algorithm [42]. Nucleotide sequence repeat elements were identified and analysed with the EMBOSS package Nucleic repeats group tools [43], and by performing a local blastn [35] query of the complete mtDNA sequence against itself. The hits were clustered as a function of similarity in CD-HIT Suite [44], and the h-cd-hit-est algorithm was run with consecutive 0.75, 0.80, and 0.90 cut-off values, using the sequence set that returned <0.001 blastn E-values in the 1 vs. 1 search.

**Table 4:** Intron-homing endonuclease domains and their location in the *P. radiata* mtDNA.

Homing endonuclease			Length (aa)			Similarity (aa% identity)			Locus distance (bp)		
Catalytic domain	Pfam	Sum	Min	Max	Mean	Min	Max	Mean	Min	Max	Mean
**LAGLIDADG-1**	CL0324	28	29	115	69	3.2	32	11	408	141 226	56 490
**LAGLIDADG-2**	CL0324	19	34	181	102	0	67	16	106	114 830	34 171
**GIY-YIG**	CL0418	10	21	112	80	12	52	26	2 214	73 172	26 106
**All**		57	28	136	84						

### Phylogenetic Analyses

Genome accessions of completely sequenced fungal mtDNAs were retrieved from NCBI Organelle Genome Resources website [19], and linked to corresponding proteomes through GenBank [45] queries. Subsequently, super alignments were generated from USEARCH [46] de-replicated proteomes with the core of Hal pipeline [47], allowing 50% of missing data. Phylogenetic trees were constructed from 44 fungal taxa and 2 019 aa remgaps super alignment first with RAxML 8.0.0 [48] with 100 rapid bootstrap repetitions and automatic model selection (-f a -d -m PROTGAMMAAUTO) using Blastocladiomycota as outgroup (best-scoring aa model was MTZOA), and with PhyloBayes 3.3f [49] using default options, 2 parallel chains were run until maxdiff was <0.1, first 100 trees were discarded as burn-in, and one in ten remaining trees were sampled for posterior consensus. The tree was rooted from mid-point. Nodes receiving ≤0.8 posterior consensus (Bayesian) or ≤80 bootstrap support (ML) were collapsed to polytomies with TreeCollapseCL4 (http://emmahodcroft.com/TreeCollapseCL.html). The trees were edited in FigTree (http://tree.bio.ed.ac.uk/software/figtree/).

### Correlation Analyses

Sequence similarity of the core domain aa-sequences from 57 HEs in the *P. radiata* mtDNA were analyzed by generating aa-sequence pairwise distance matrix of the LAGLIDADG 1 and 2, and GIY-YIG catalytic ORFs using Geneious 5.5.5 software. In addition, pairwise distance matrices of the HE domain loci were calculated using the R environment 2.14.1 package for Windows (http://www.r-project.org/) in order to test correlation of the locus distance to the sequence-similarity based (evolutionary) distance. The data matrices were tested for being parametric or non-parametric. LAGLIDADG 1 aa-sequence similarity scores were normalized with logarithmic transformation. Parametric Pearson correlation in PASW Statistics 18, release 18.0.0 (SPSS Inc., Chicago, IL, USA) was used for LAGLIDADG 1, and GIY-YIG type HE domains, when the non-parametric Spearman’s correlation test was applied to LAGLIDADG 2.

## Results

### *P. radiata* mtDNA Genome Structure and Conserved Genes

**Figure 1:**
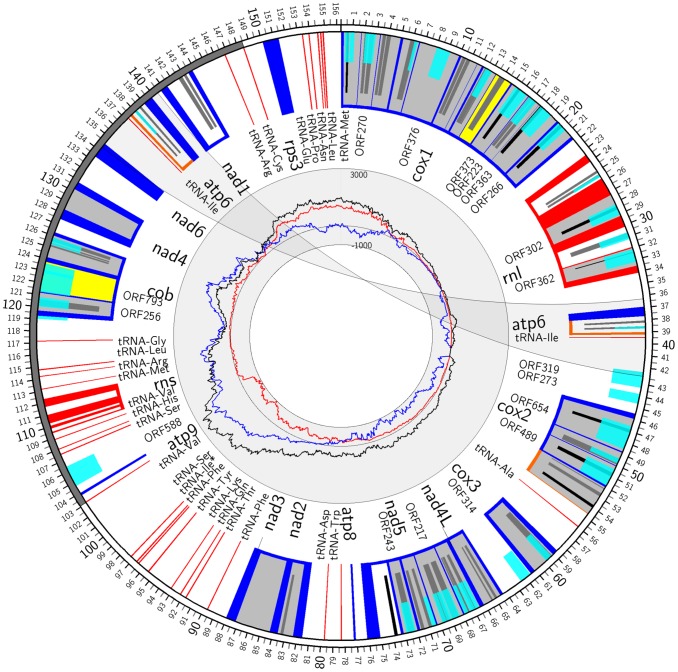
Gene map of *Phlebia radiata* mtDNA. Colour of the scale (kb) bar indicates orientation of transcription: clockwise (CW, white), counter-clockwise (CCW, grey). Bars mark protein-coding (blue) and RNA (red) genes, and alternative C-termini in *atp6* and *cox2* are depicted (orange). Intron type is indicated in colour: group I (light grey), group II (yellow), and uncertain (white). Within introns, the hypothetical and identified ORFs are indicated: over >200 amino-acid long ORFs (turquoise), and homing endonuclease domains GIY-YIG (black) and LAGLIDADG (dark grey). The transparent ribbon illustrates location of the 6,076 bp inversion-duplication. Asterisk indicates putative tRNA-Ile^CAU^. The inner circle (scale at 12 o’clock in linear units) plots nucleotide bias (G/C skew) up to hexamers along each mtDNA position; G/C (red), A/T (blue), total strand bias (black), placing *oriC* around 11∶30 o’clock as the largest bias of G over C.

The mitochondrial genome (mtDNA) of *Phlebia radiata* isolate 79 was achieved by *de novo* 454 sequencing of total DNA using Titanium chemistry, and the final assembly resulted in a single 156 348 bp scaffold with a sequence coverage of over 300x, representing one circular dsDNA molecule (Figure 1) with a GC percentage of 31.1. The genome contains the 14 protein-coding genes typical to fungal mtDNA, which are related to the mitochondrial inner membrane Complexes I, III, IV and V of the respiratory chain, i.e. *cox1, atp6, cox2, cox3, nad4L, nad5, atp8, nad2, nad3, atp9, cob, nad4, nad6, nad1*, in clockwise order of the mtDNA (Figure 1). Additionally, 31 conserved genes related to information transfer (28 tRNAs, *rnl*, *rns*, and *rps3*) were identified (Tables 1, 2).

**Table 1:** Annotated conserved protein-coding and rRNA genes, their characteristics, location and intron types in *P. radiata* mtDNA.

Gene	Start	End	Strand	Length bp	Coding sequence length (bp)	Protein length (aa)	Introns			Average intron length (bp)	Coding sequence density	Stop codon
							group I	group II	un-certain			
*cox1*	1	21743	+	21743	1590	529	12	1		1550	7.3%	TAA
*rnl*	23389	34087	+	10699	3624		2		2	1769	33.9%	
*atp6*	36924	37700	+	777	777	258					100.0%	TAA
*cox2*	45480	52022	+	6543	756	251	3			1929	11.6%	TAA
*cox3*	58610	61439	+	2830	813	270	1			2017	28.7%	TAA
*nad4L*	64304	66152	+	1849	273	90	1			1576	14.8%	TAA
*nad5* [1]	66153	76178	+	10026	2007	668	4		1	1604	20.0%	TAA
*atp8*	77128	77286	+	159	159	52					100.0%	TAA
*nad2*	81572	87656	+	6085	1812	603	2			2137	29.8%	TAA
*nad3* [2]	87656	88030	+	375	375	124					100.0%	TAA
*atp9*	103782	104003	−	222	222	73					100.0%	TAA
*rns*	109435	112341	−	2907	1711				3	399	58.9%	
*cob* [3]	119244	126306	−	7063	1272	423	2	1		1930	18.0%	TAA
*nad4*	127498	130848	−	3351	1473	490	1			1878	44.0%	TAA
*nad6* [4]	133402	134382	−	981	981	326					100.0%	TAA
*atp6*	139006	139782	−	777	777	258					100.0%	TAA
*nad1* [5]	140738	143807	−	3070	1017	338			1	2053	33.1%	TAG
*rps3*	150069	151403	+	1335	1335	444					100.0%	TAA

[1] *nad5* starts from the adjacent in frame codon to *nad4L* stop.

[2] *nad3* uses the last A of *nad2* stop codon for initiation Met’s first nt.

[3] Based on multiple sequence alignment of *Basidiomycota cob* genes the last conserved aa of *P. radiata cob* is 43 aa before the stop codon.

[4] Based on multiple sequence alignment of *Basidiomycota nad6* genes the last conserved aa of *P. radiata nad6* is 122 aa before the stop codon.

[5] The codon after the putative TAG stop codon is TAA.

Empty cell, not present or observed, or unable to calculate.

**Table 2:** Transfer RNA genes in *P. radiata* mtDNA.

Transfer RNA[1]	Anticodon	Start	End	Strand	Length (bp)
Ile	GAU	39443	39514	+	72
Ala	UGC	55927	55999	+	73
Trp	CCA	78296	78369	+	74
Asp	GUC	79686	79758	+	73
Phe	GAA	89697	89767	+	71
Thr	UGU	91722	91793	+	72
Gln	UUG	92238	92311	+	74
Lys	UUU	93159	93231	+	73
Tyr	GUA	95474	95557	+	84
Phe	GAA	96533	96603	+	71
Ile[2]	CAU	96632	96704	+	73
Ser	UGA	97243	97328	+	86
Val	UAC	103081	103151	−	71
Ser	GCU	107528	107609	−	82
His	GUG	107979	108050	−	72
Val	UAC	108850	108920	−	71
Met	CAU	113144	113216	−	73
Arg	UCG	114191	114261	−	71
Leu	UAG	114743	114816	−	74
Gly	UCC	117118	117188	−	71
Ile	GAU	137192	137263	−	72
Arg	UCU	146748	146818	−	71
Cys[3]	GCA	148390	148461	−	72
Pro	UGG	153357	153429	+	73
Asn	GUU	153900	153972	+	73
Leu	UAA	154617	154701	+	85
Met	CAU	154736	154807	+	72
Glu	UUC	154832	154902	+	71

[1] tRNAscan predicts the tRNA type from the anticodon.

[2] This tRNA was determined to be Ile through comparative means (see below).

[3] The bit score of tRNA-Cys was below 20, which is a typical cut-off value for a pseudogene. The gene was predicted with exceptionally low score from all Basidiomycota mtDNAs.

**Figure 2:**
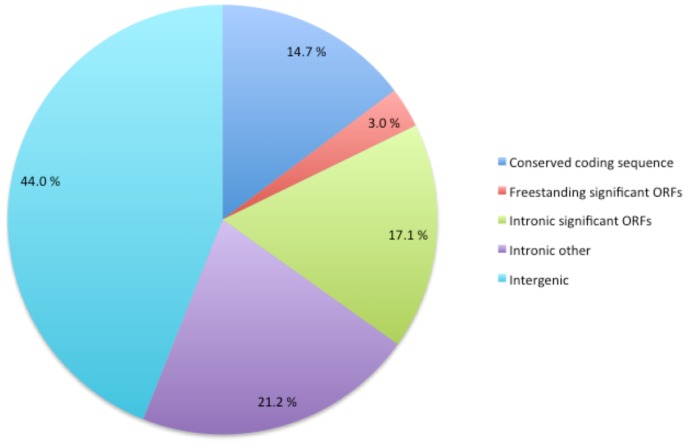
Contribution of the various features of *P. radiata* mtDNA to genome size. Conserved coding sequence refers to the conserved fungal mitochondrial proteome ORFs, rRNAs and tRNAs. Significant ORFs refer to additional identified and hypothetical protein coding sequences with E-value<0.001 obtained by BLASTp queries. Freestanding refers to intronless. Intronic significant ORFs include homing endonucleases, also exon-to-exon-fused ORFs, excluding intergenic ORF HEGs (see text).

Identified protein (sum 68 953 bp), rRNA (sum 13 606 bp), and tRNA (sum 2 070 bp) genes including introns cover 55% of the *P. radiata* mtDNA. However, only about 15% (25 045 bp) of the genome refers to conserved coding sequences, when intergenic regions (in total 44% of the genome) and coding-sequence splicing introns (sum 59 584 bp, 38%) dominate the sequence space (Figure 2). The majority of the conserved protein-coding genes were split by long introns into multiple short exons (Figure 1, Table 1). The highest number of introns (13) was in the *cox1* gene, which covered ca. 21 kbp (14%) of the genome.

**Figure 3:**
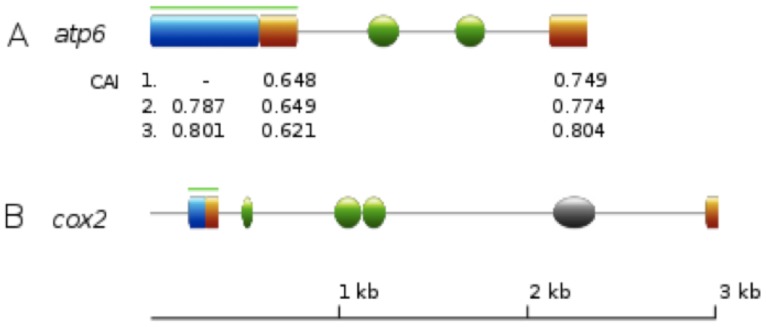
Schematic view of the C-termini regions of *P. radiata* mtDNA *atp6* and *cox2* genes. Green lines denote *atp6* coding sequence region and the last exon of *cox2*. Spheres/ovals represent LAGLIDADG 1 (green) and GIY-YIG (grey) homing endonuclease domains. **A)** Region of 201 bp (orange) with high 1 vs. 1 sequence similarity, corresponding to the 3′-end of the *atp6* gene. Codon Adaptation Index (CAI) values are shown for the *atp6* N-terminal region (blue) and for the regions of high sequence similarity. Reference codon usage is from: 1) *atp6* N-terminus, 2) *atp6* N-terminus and *atp8*-*9*, 3) *atp6* N-terminus, *atp8-9*, *cox1-3*, *nad1-5* and *nad4L*. **B)** Separated by three LAGLIDADG 1 domains and a GIY-YIG domain, two regions of high 1 vs. 1 sequence similarity (orange) exist for the last 66 bp of the *cox2* gene. Image was generated with ExPASy PROSITE MyDomains Image Creator (http://prosite.expasy.org/mydomains/) and edited in Inkscape version 0.48.2 (http://inkscape.org/).

Notably, the genes encoding *atp6*, tRNA-Ile^GAU^ and tRNA-Phe^GAA^ were present in two identical copies. The duplicate *atp6* and tRNA-Ile^GAU^ genes were identified in the “mirror” region, which comprised an inverted and almost identical 6.1 kbp region in the genome (Figure 1). On the basis of multiple sequence alignments, *cob* and *nad6* ORFs had C-terminal fused extensions. Moreover, alternative 3′-ends were found for *atp6* and *cox2* (Figure 3 A, B).

### Open Reading Frames with Unknown or Non-conserved Function

In total, 108 ORFs in addition to the conserved genes met our initial search criteria (Table S1). From these, 39 produced significant (E-value ≤0.001) blastp hits against the nr database, with a Codon Adaptation Index (CAI) range of 0.299–0.800 in reference to the conserved protein-coding genes. The majority of these ORFs were intronic and were associated with HE domains (Table 3). A notable exception was ORF793 within the long group II intron in the middle of *cob* gene (Figure 1). This intronic ORF was associated with identified RNA-dependent DNA polymerase domain (annotated locus PRA_mt0165, reverse transcriptase) and had particularly low CAI-value of 0.299 (Table S1), which indicates relatively recent horizontal gene transfer from a genetically distant source, most probably of viral origin.

**Table 3:** Introns, their lengths and types in the conserved *P. radiata* mtDNA-encoded genes.

Gene	Intron 1	Intron 2	Intron 3	Intron 4	Intron 5	Intron 6	Intron 7	Intron 8	Intron 9	Intron 10	Intron 11	Intron 12	Intron 13	Total length (bp)	Average length (bp)
*cox1*	1463 G	1540 L1	1680 L1	1356 L2	3420	1447 L1	432	1144 L2	1410 L2	1649 G	1076 L1	2004 G	1532 G	20153	1550
*nad5*	1413 L1	2278 L2	1405 L1	1186 L2	1737 G									8019	1604
*rnl*	1675 L2	1912 G	1756 L2	1732 G										7075	1769
*cox2*	2849 G	1152 L2	1786 G											5787	1929
*rns*	704	201	291											1196	399
*cob*	1864 L1	2452 R	1475 L2											5791	1930
*nad2*	1306 L2	2967												4273	2137
*cox3*	2017 L2													2017	2017
*nad4L*	1576 L1													1576	1576
*nad4*	1878													1878	1878
*nad1*	2053 L1													2053	2053

Homing endonuclease associations: G, GIY-YIG; L1, LAGLIDADG 1; L2, LAGLIDADG 2; R, reverse-transcriptase ORF including.

Underlined, group II intron type.

Due to annotated genome sequence submission requirements, ORFs that continued from undisrupted exon reading frames into putative intronic regions were 5′-truncated to their first Met codons, which shortened eight annotated ORFs, and excluded five ORFs that returned significant E-values (Table S2). These ORFs may represent inteins (“protein introns”).

Freestanding *P. radiata* mtDNA ORFs that returned significant blastp hits were ORF588, ORF319, ORF314, ORF273 and ORF90, with respective CAI values of 0.624, 0.577, 0.747, 0.633, and 0.515 (Table S1), indicative of fungal mitochondrial origin. Two of these, ORF588 (PRA_mt0150) and ORF273 (PRA_mt0076), were the most similar to putative DNA polymerases of *Pleurotus ostreatus* mt genome (Table S1). Two coding sequences, ORF319 and ORF314, were the most similar to hypothetical proteins annotated in *Moniliophthora roreri* mtDNA as orf2 and hyp11, respectively. Notably, the 5′-end of ORF319 is similar to that of the *P. radiata* mt *nad6* gene (36/37 nt identities), as it is located at the edge of the mirror region (Figure 1). The best hit for ORF90 in turn was a hypothetical protein annotated in the mtDNA of the Ascomycota species *Ajellomyces dermatitidis.*

### Transfer RNAs and Codon Usage

The tRNAscan-SE algorithm identified 28 tRNAs (Table 2). This tRNA set is likely able to sense all the codons of the *P. radiata* mtDNA-encoded proteome. With the exception of anticodons of Trp and Ile tRNAs, where possible, U was always the anticodon wobble position base. In the remaining tRNAs, G was always used over A at the anticodon wobble position. For the tRNA-Cys, the tRNAscan-SE Cove algorithm predicted probability for a gene match score below the threshold value of 20.0. However, Cove scores were low as well for other Basidiomycota tRNA-Cys genes, e.g. in *Phakopsora meibomiae* (cove: 19.75), *P. ostreatus* (cove: 19.67), and *Schizophyllum commune* (cove: 22.36). This indicates that the *P.radiata* mtDNA tRNA-Cys gene is real despite the low Cove score obtained.

The GC-content of *P. radiata* mtDNA ORFs was 26.83% (1st letter GC: 34.14%, 2nd letter GC: 33.33%, 3rd letter GC: 13.00%), with no obvious bias observed in codon usage between the leading and the lagging strand encoded ORFs. With the exception of Trp, W-base (A or T/U) ending codons were preferred over S-ending (C or G) codons across all codon families. Cys codons showed the smallest bias with 72% UGU over 28% UGC. For Ala, Phe, His, Ile, Asn, Pro and Tyr the same percentage was ≥80%, and for the rest ≥90%. Some codons UGA (1), AAG (9), CGC (1), AGG (5), and CGG (2) may be unassigned, as they were only present in non-conserved regions, mainly in the putative non-translated C-terminal fused extensions of *cob* and *nad6*.

### Phylogeny

**Figure 4:**
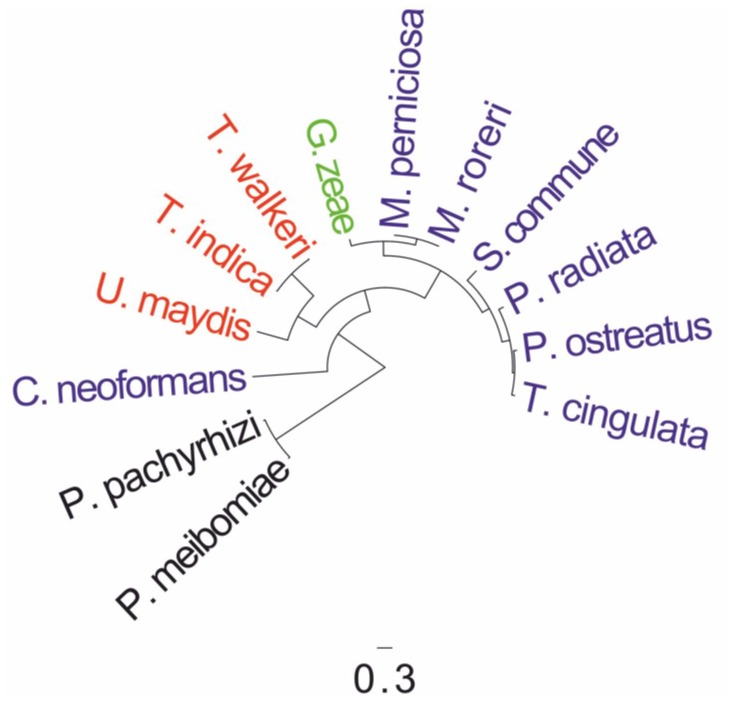
Codon usage of the *P. radiata* mitochondrial genome protein-coding gene open reading frames in comparison to selected Basidiomycota and Ascomycota species. Neighbor-joining tree with topology derived from codon on codon root mean squared difference distance matrix (Table S3, Table S4). Agaricomycotina, blue; Ustilaginomycotina, red; Pucciniomycotina, black; Ascomycota species *Gibberella zeae = Fusarium graminearum*, green, as outgroup. Scale bar indicates nucleotide changes per site.

The first phylogenetic analysis was established on the similarity and variations of codon usage in fungal mtDNA-protein coding sequence ORFs (Figure 4). The restricted amount of species included in the analysis, however, already grouped *P. radiata* mtDNA-proteome with Agaricomycotina. Exceptional was the positioning of *C. neoformans* far out from the other Agaricomycotina species.

**Figure 5:**
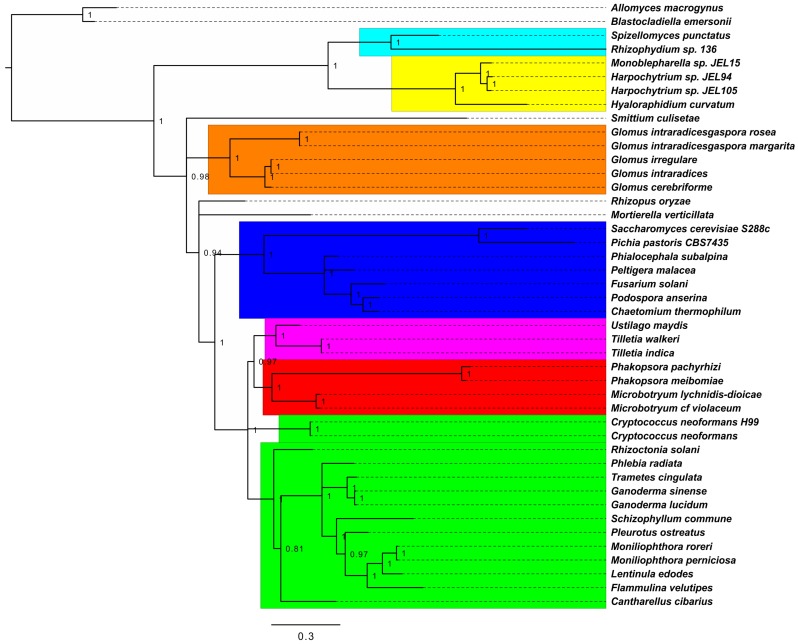
Phylogeny of fungal mitochondrial proteomes. The statistically most likely tree was derived by Bayesian inference from a multi-gene superalignment of mtDNA-encoded proteins (2 019 aa positions, 44 taxa). Posterior consensus support values are depicted for branching, and nodes receiving ≤0.8 support were collapsed into polytomies. The tree was rooted from mid-point. Colours refer to phyla or sub-phyla: turquoise, Chytridiomycota; yellow, Monoblepharidomycota; orange brown, Glomeromycota; dark blue, Ascomycota; pink, Ustilaginomycotina (Basidiomycota); red, Pucciniomycotina (Basidiomycota); bright green, Agaricomycotina (Basidiomycota); Blastocladiomycota node as outgroup. Scale bar indicates amino-acid substitutions per site. For species and mtDNA accession, see Table 5.

Our protein phylogenetic approaches, the maximum-likelihood based RAxML, and the Bayesian Monte Carlo Markov Chain sampler PhyloBayes, reconstructed the recognized fungal phyla as monophyletic clades. However, in RAxML the branching of Chytridiomycota, Glomeromycota, and Dikarya (Ascomycota and Basidiomycota) was polytomic, whereas in the PhyloBayes derived tree, Chytridiomycota and Monoblepharidomycota were a sister lineage to Glomeromycota and Dikarya, and Glomeromycota were a sister lineage to Dikarya (Figure 5). Further, in the Bayesian phylogeny, the *incertae sedis* species (previous Zygomycota) *R. oryzae* and *M. verticillata* were within the Glomeromycota/Dikarya branch.

Basidiomycota subphyla were resolved by both methods to current fungal taxonomy with the single exception of the Agaricomycotina classified *C. neofarmans* node (two strains, Figure 5). As with higher-level taxonomy, PhyloBayes seemingly solved Basidiomycota subphylum level phylogeny with less polytomies, placing Agaricomycotina as a sister lineage to the Pucciniomycotina/Ustilaginomycotina group. *P. radiata* positioned nearest to *Trametes cingulata* and two *Ganoderma* species (Figure 5).

### Introns and Intron Homing Endonucleases

Nine of the 16 fungal mitochondrial conserved genes in *P. radiata* mtDNA were interrupted by over 1 000 bp long introns (Table 3). RNAweasel [42] algorithm detected 29 group I and two group II intron structures, out of which all but one were located within regions that were determined to be intronic also by our manual approach (blastp, blastn, Pfam queries, ClustalX alignments). Our semi-manual approach (blastp, blastn, Pfam queries, ClustalX alignments) predicted seven additional introns. Four of these were associated with core catalytic HE domains within the *nad1*, *nad2* and *rnl* gene regions. Despite the lack of sequence homology, three shorter introns were inferred to reside within the *rns* gene (Table 3). In total, 29 introns were associated with HE domains. Introns varied in length from 201 bp (intron 2 in *rns*) to 3 420 bp (intron 5 in *cox1*), with an average length of about 1.5 kbp for the protein-coding gene splicing introns (Table 3).

Based on Pfam queries, the *P. radiata* mtDNA contained 57 characteristic protein domains encoded by HE genes (HEGs, Pfam family PF05204; Table 4) belonging to three different structural families: LAGLIDADG (47) with subtypes 1 (28) and 2 (19), and GIY-YIG (10). The catalytic HE domains expanded from 21 to 181 aa (59 to 542 bp), and their aa pairwise sequence similarities varied from 3.2% to 32% for LAGLIDADG 1, from no identity to 67% similarity for LAGLIDADG 2, and from 12% to 52% for GIY-YIG type of domains (Table 4, Table S5). The HE motifs were predominantly (45/57) situated within group I type introns. One single GIY-YIG domain located within a group II intron, and 10 motifs located in regions of unrecognized intron type (Table 3).

Eight of the HE catalytic domains were exceptional in appearing as free-standing after the last putative coding sequence exon and stop codon in the genes *atp6* and *cox2*. Notably, alternative C-termini were annotated for both of these genes (Figure 3A, B). The parametric Pearson’s correlation test of pairwise aa-similarity and locus distance for the identified HE domain types (Table S1, S2, S5) resulted in correlation value of −0,166 with a statistically significant p-value (0.031) for LAGLIDADG type 1, and correlation value of −0.256 with, however, a statistically insignificant p-value (0.089) for GIY-YIG type. The non-parametric Spearman’s correlation test for LAGLIDADG type 2 (19) resulted in a test value of −0.040 but with statistically insignificant p-value (0.607). These results infer that genome (intron) location is to some extent related to the degree of HE sequence similarity, at least for the LAGLIDADG 1 HEs. However, it may also be concluded that HE motif transposition to more distant locations are equally allowed, as is observed for LAGLIDADG 2 and GIY-YG domains.

### Inverted Duplication and Other Repeated Elements

A distinguishing feature was the inverted duplication region of 6 075 bp in size that accounted for 3.9% of the *P. radiata* mtDNA. One region (ID1) expanded from nt position 140 421 to 134 346 and the other (ID2) from nt position 36 285 to 42 360 (Figure 1), which is named “mirror region” in our EMBL submitted and annotated *P. radiata* mt genome. Both regions harboured the two genes *atp6* and tRNA-Ile^GAU^, and differed only by 3 nt –in the plus DNA strand at nt position 40 066 with an additional A, at nucleotide position 41 338 with absence of T, and at position 42 353, T instead of G.

**Figure 6:**
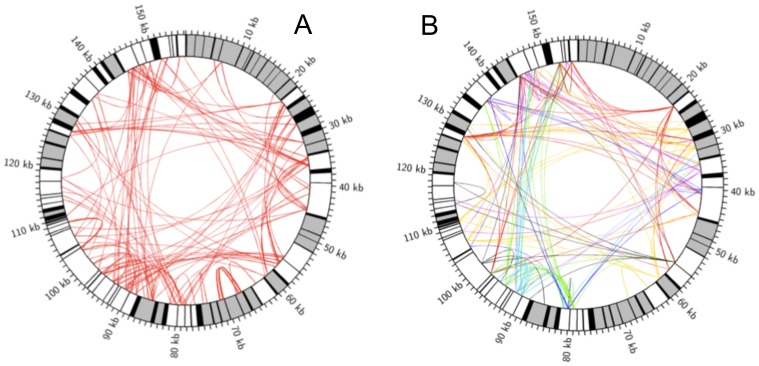
Dispersed and inverted repeat sequences in *P. radiata* mtDNA. Colors: black, conserved protein-coding, rRNA and tRNA genes; grey, introns; white, intergenic regions. The large 6.2 kb duplication-inversion is excluded from the figures. **A)** Red ribbons connect regions of significant (E-value <1×10^−7^) nucleotide sequence similarity. **B)** Coloured ribbons connect similar sequence regions. Only clusters with at least 6 similar repeat members are shown. The sequences were clustered as a function of similarity in CD-HIT Suite [44] (h-cd-hit-est run with consecutive 0.75, 0.80, and 0.90 cut-off values) from a sequence set that returned <0.001 1 vs. 1 blastn values.

A major difference between the ID1 and ID2 regions was the start of the coding sequence of the single-copy gene *nad6* within the 3′ end of the ID1 region (Figure 1). Another difference was the occurrence of a single-copy, functionally unknown ORF319 (PRA_mt0074) that was only recognized in ID2. However, the ORF was only 1-nt different in the 5′ end sequence (first 37 nt) compared to *nad6* in ID1. In addition to the mirror region, the *P. radiata* mtDNA is frequent with short (≤200 nt), dispersed, and partially overlapping tandem repeat sequence motifs (Figure 6A, B), in particular between positions 85 000 to 100 000 nt, where also tRNA encoding genes were clustered (Figure 1). The most abundant repeat sequence types were dispersed and inverted repeat sequences, which were almost exclusively localized into intronic, and especially into intergenic regions (Figure 6A). These repeats were often overlapping, and covered as much as 15% of the *P. radiata* mtDNA. Subsets of these sequences shared high sequence similarity (Figure 6B).

### Origin of Replication

According to G/C skew analysis, origin of replication (*oriC*) of the *P. radiata* mtDNA may be located around 11∶30 to 00∶30 o’clock (position 153 000 to position 7 nt) regarding to the largest bias of G over C (Figure 1). The lowest G/C skew ratio in turn is located around 7∶00 to 8∶30 o’clock (positions 88 000 to 109 000 nt), in the about 21 kb size intergenic region indicative of a putative mtDNA replication termination site, which is supported by switching of the coding strand (orientation of transcription) at this site, around position 103 000 nt.

### Sequence Accession

The complete and annotated *Phlebia radiata* 79 mitochondrial genome sequence is available under the accession codes [EMBL: HE613568] and [NCBI: NC_020148].

## Discussion

### mtDNA Size and Genome Organization

To our knowledge, at 156 348 bp, the mitochondrial genome of *P. radiata* described in our current study is the second largest completely sequenced, gene annotated and located mtDNA among fungi, and presents specific features as signs of genetic flexibility, recombination history and active editing process of the genome. Our findings on the size and original features of the *P. radiata* mtDNA, together with other recent Basidiomycota mitochondrial genome studies are thereby not explicitly supporting the previous conclusions for the rather small sized and disappearing fungal mitochondrial genomes.

On the contrary, fungal mt genomes apparently vary greatly in size, from ca. 12 kb kbp of the Cryptomycota parasite species *Rozella allomycis* [19] to over 235 kbp (165 kbp main mtDNA [50]) of the Basidiomycota Agaricomycotina species *Rhizoctonia solani* strain AG3 Rhs1AP (Table 5). Evidence of large variations in the genome size and a high degree of mtDNA structural complexity between eukaryotic organism lineages and species is currently accumulating through sequencing projects. For fungi, such extreme variations in the mtDNA size or multi-chromosomal organization have not, however, been noticed than is updated for plant (from 0.2 to 10 Mbp mtDNAs), algae and protozoa mitochondria [18]–[22]. Fungal mitochondrial genomes are usually mapped as single circular dsDNA molecules [7], [8], [23]–[29]. We likewise assume a similar configuration for the *P. radiata* mtDNA on the basis of our sequence assembly, bioinformatic analyses and PCR. Reports on more linear than circular chromosomal structure of the mtDNA in the Chytridiomycota species *Hyaloraphidium curvatum* [30], in the Ascomycota yeasts *Candida albicans* [31] and *Saccharomyces cerevisiae* [32], [51], [52] indicate that the possibility for a partial linear or linear-circular chromosomal organization for the *P. radiata* mtDNA cannot be completely ruled out, despite the convincing circular assembly which was obtained from the careful study of our sequence data.

**Table 5:** Fungal mitochondrial genomes, representatives of Basidiomycota and other phyla. *Phlebia radiata* mtDNA, this study; *R. solani 162 751 kbp [50], 235 849 kbp [19].

Fungal phylum	Species	Subphylum or class	mtDNA sequence accession	mtDNA size (bp)
**Basidiomycota**				
	*Rhizoctonia solani*	Agaricomycotina	NC_021436	*162 751
	***Phlebia radiata***	Agaricomycotina	**NC_020148**	156 348
	*Agaricus bisporus*	Agaricomycotina	JX271275	135 005
	*Lentinula edodes*	Agaricomycotina	NC_018365	121 394
	*Moniliophthora perniciosa*	Agaricomycotina	NC_005927	109 103
	*Moniliophthora roreri*	Agaricomycotina	NC_015400	93 722
	*Trametes cingulata*	Agaricomycotina	NC_013933	91 500
	*Flammulina velutipes*	Agaricomycotina	NC_021373	88 508
	*Ganoderma sinense*	Agaricomycotina	NC_022933	86 451
	*Pleurotus ostreatus*	Agaricomycotina	NC_009905	73 242
	*Ganoderma lucidum*	Agaricomycotina	NC_021750	60 630
	*Cantharellus cibarius*	Agaricomycotina	NC_020368	58 656
	*Schizophyllum commune*	Agaricomycotina	NC_003049	49 704
	*Cryptococcus neoformans var. grubii H99*	Agaricomycotina	NC_018792	24 919
	*Cryptococcus neoformans var. grubii*	Agaricomycotina	NC_004336	24 874
	*Tilletia indica*	Ustilaginomycotina	NC_009880	65 147
	*Tilletia walkeri*	Ustilaginomycotina	NC_010651	59 352
	*Ustilago maydis*	Ustilaginomycotina	NC_008368	56 814
	*Microbotryum lychnidis-dioicae*	Pucciniomycotina	NC_020353	107 808
	*Microbotryum cf. violaceum*	Pucciniomycotina	NC_020354	92 107
	*Phakopsora meibomiae*	Pucciniomycotina	NC_014352	32 520
	*Phakopsora pachyrhizi*	Pucciniomycotina	NC_014344	31 825
**Ascomycota**				
	*Chaetomium thermophilum var. thermophilum*	Pezizomycotina	NC_015893	127 206
	*Podospora anserina*	Pezizomycotina	NC_001329	100 314
	*Fusarium graminearum (Gibberella zeae)*	Pezizomycotina	NC_009493	95 676
	*Peltigera malacea*	Pezizomycotina	NC_016955	63 363
	*Fusarium solani*	Pezizomycotina	NC_016680	62 978
	*Phialocephala subalpina*	Pezizomycotina	NC_015789	43 742
	*Nakaseomyces bacillisporus*	Saccharomycotina	NC_012621	107 123
	*Saccharomyces cerevisiae*	Saccharomycotina	NC_001224	85 779
	*Komagataella (Pichia) pastoris*	Saccharomycotina	NC_015384	35 683
**Glomeromycota**				
	*Gigaspora rosea*	Glomeromycetes	NC_016985	97 350
	*Gigaspora margarita*	Glomeromycetes	NC_016684	96 998
	*Glomus irregulare*	Glomeromycetes	NC_014489	70 800
	*Glomus (Rhizophagus) intraradices*	Glomeromycetes	NC_012056	70 606
	*Glomus cerebriforme*	Glomeromycetes	NC_022144	59 633
**Chytridiomycota**				
	*Spizellomyces punctatus*	Chytridiomycetes	NC_003052	61 347
	*Rhizophydium* sp. 136	Chytridiomycetes	NC_003053	68 834
**Monoblepharidomycota**				
	*Monoblepharella* sp.	Monoblepharidomycetes	NC_004624	60 432
	*Hyaloraphidium curvatum*	Monoblepharidomycetes	NC_003048	29 593
	*Harpochytrium* sp.	Monoblepharidomycetes	NC_004623	24 169
	*Harpochytrium sp.*	Monoblepharidomycetes	NC_004760	19 473
**Blastocladiomycota**				
	*Allomyces macrogynus*	Blastocladiomycetes	NC_001715	57 473
	*Blastocladiella emersonii*	Blastocladiomycetes	NC_011360	36 503
**Cryptomycota**				
	*Rozella allomycis*		NC_021611	12 055

With fungal mt genomes of less than 30 kb in size, usually all the 14 fungal mtDNA-conserved, mitochondrial inner-membrane protein complex I, III, IV and V protein subunit-coding genes are present. Examples of these compact mt genomes within Basidiomycota are species of the animal-pathogenic genus *Cryptococcus*, with some variation of the mtDNA size (24–34.7 kb), gene order and intronic and ORF coding sequence between species and variants [53], [54]. The large fungal mitochondrial genomes, alike *P. radiata* mtDNA, expand over 100 kb in size and may include over 50 protein-coding ORFs (Table 5). The tendency for larger fungal mt genomes (over 90 kbp) in the Basidiomycota subphylum Agaricomycotina is pinpointed by recent reports on *Moniliopthora perniciosa* and *M. roreri* [24], [28], *T. cingulata* (over 90 kb) [26], *A. bisporus* (135 kbp) [23] and *R. solani* (165 kbp/235 kbp) [50]. This tendency is furthermore confirmed by our study on the 156 kbp *P. radiata* mtDNA showing up to 126 predicted protein-coding ORFs and 30 RNA genes, which are the second highest numbers reported for the fungal mitochondrial genomes.

The largest mtDNAs within Ascomycota are those of the filamentous species *Podospora anserina* (over 100 kbp) and *Chaetomium thermophilum* var. *thermophilum* (over 120 kbp), similar to many of the Agaricomycotina basidiomycete mtDNAs, when the smallest (from Ascomycota yeasts) mt genomes are reduced to about 20 kbp in size (Table 5) with only 10 protein-coding genes [19]. Among Ascomycota fungi, however, there are yet large variations at the genus level – between species - of both mtDNA size and gene content. In the yeast *S. cerevisiae*, the 85.8 kbp mtDNA harnesses 19 protein-coding genes when only the mitochondrial Complex I NADH dehydrogenase subunit encoding genes are absent [19], [29], [51]. In another species of *Saccharomyces, S. castellii*, the mtDNA is reduced to 1/3 in size (25.7 kb) and contains only 9 protein-coding genes [51].

Only slight differences, however, in mtDNA size, gene number and organisation have been observed in the Basidiomycota genera *Moniliophthora* (Agaricomycotina) [24], [28], *Tilletia* (Ustilaginomycotina) [19], *Phakopsora* (Pucciniomycotina) [27], and *Cryptococcus* (Agaricomycotina) [53], [54], thus indicating genus-level conservation of the mitochondrial genomes in the phylum Basidiomycota. The large differences in gene order and location (loss of synteny) between the Basidiomycota mitochondrial genomes at higher taxon levels, as was reported for *Ganoderma lucidum* (Agaricomycotina) mtDNA [55], and is observed in this study for *P. radiata* mtDNA, may thus indicate frequent recombination events and flexibility of fungal organelle genomes.

### Intergenic and Repetitive Sequences

In the large angiosperm plant mt genomes, the genome size is mainly due to long intergenic regions and non-coding sequence (gene spacers, introns, and pseudogenes) [20], [22]. Accordingly in the large *P. radiata* mtDNA, over 80% of the mt genome is either intergenic or intronic sequence (Figure 2). Our analyses show that most of the intergenic sequence space in the *P. radiata* mtDNA is filled with repeated sequences, in particular between genome nt positions from 90 000 to 110 000 surrounding several tRNA-coding gene loci. Accumulation of polymorphic microsatellite repeated elements (1–6 nt in length) were reported for species of the Agaricomycotina genus *Agrocybe* [56], and already in *S. cerevisiae* mtDNA, long AT rich stretches were identified [29]. Putative roles in splicing of mitochondrial polycistronic transcripts may be proposed for the intergenic regions, as well as action as potential promoters with regulative element motifs.

Surprisingly, the mtDNA of *P. radiata* contains a large (6.1 kbp) inverted duplication segment. This is another similar feature to the angiosperm plant mitochondrial genomes, where large duplicated sequences in the mtDNA apparently function as co-linear recombination sites to aid genome organization [21], [22]. In the Ascomycota species *C. albicans*, the very similar in size (7 kbp) inverted repeat regions in the apparently linear mtDNA are directing genome replication *via* homologous recombination at the site [31]. Interestingly, the *C. albicans* mtDNA inverted repeat harnesses duplication of the gene *cox3*, whereas in the *P. radiata* mtDNA mirror site, we recognized duplication of the genes *atp6* and tRNA-Ile^GAU^. Two inverted repeats of over 4 kbp in size were reported for another Agaricomycotina species, *A. bisporus,* devoid of protein-coding ORFs but containing duplicated sets of tRNA genes [23]. At the moment, we may suggest a replication-directing recombination function for the inverted duplication in the *P. radiata* mt genome, similar to that observed in *C. albicans* mtDNA [31].

### Introns and Homing Endonucleases

Together with the high portion of the intergenic regions, notable in *P. radiata* mtDNA is the degree of invasion by mobile DNA-elements, which were recognized as long type I and II self-splicing introns (in total over 30 long introns), and including up to 57 HE domain-encoding ORFs. Nine of the 15 conserved protein-coding genes, and the *rnl* gene in *P. radiata* mtDNA are invaded by long introns carrying HE motifs of LAGLIDADG types I and II, when the ten recognized GIY-YIG motifs correspond a minority of the HE domain types.

Homing endonuclease genes were previously identified within mtDNAs of other Basidiomycota species, such as *M. perniciosa* and *M. roreri* [24], [28] and *A. bisporus* [23]. However, only four of the protein-coding genes (*cob*, *cox1,2*, and *nad5*), and the *rns* and *rnl* genes were previously reported to contain self-splicing introns with HE motifs in these fungal mtDNAs, when in the *P. radiata* mtDNA, nine of the 11 intron-containing conserved genes contained intronic HE domains. In the elongated *cox1* gene of *P. radiata*, eleven LAGLIDADG and four GIY-YIG motifs were recognized in 13 long, self-splicing type I (12) and type II (1) introns. In regard to this, even up to 18 introns, both type I and II, were recognized in the almost 30 kb-size *cox1* gene of *A. bisporus* [57].

A few reports enlighten the enzymatic and molecular functions of intronic HEs in the fungal mtDNAs. In gene transcription, the HE domains are apparently removed from the transcribed pre-mRNA resulting in a contiguous RNA transcript [58]–[60]. It is likely that existence of intron-homing endonucleases within fungal mtDNA genes is one apparatus for promoting genetic diversity and adaptive response for the mitochondrial genome, when the allelic recombination events may be impossible or rare due to the mainly uniparental nature of mtDNA inheritance in fungi [10], [17].

In the Ascomycota species *Aspergillus nidulans*, C-terminal fragment of the mtDNA *cob* gene intron-homing translated LAGLIDADG type endonuclease (I-AniI) is involved in splicing of the intronic pre-mRNA, while the second, N-terminal endonuclease motif was not essential for intron splicing [58]. Instead, the N-terminal motif functioned in cleavage of the DNA target site to initiate the HE intron mobilization. These reports on well-ordered and bi-functional effects of the intronic HEs imply their active involvement in supporting genetic flexibility in the fungal mitochondrial genomes.

The HEs recognize longer DNA target regions (14–40 bp) than common DNA-endonucleases and tolerate more sequence variation, which assists in interrupting and introducing new genetic elements (usually introns) and ORFs (mainly intronic HE domains) in their target sites [59], [60]. In *P. radiata* mtDNA, two potential examples (genes *atp6* and *cox2*) of HE-transmitted introns and alternative coding sequences, both C-terminal, are observed (Figure 3A, B). Unusual splicing of the mtDNA *atp6* C-terminus was early on reported for the Blastocladiomycota species *Allomyces macrogynus* [61]. Additional intron with HE was found as an insertion including foreign C-terminal *atp6* sequence fused in-frame, which was explained by horizontal gene transfer [61]. However, a more likely explanation is that intronic HEs may have transposed to a new site downstream of the stop codon, as has apparently occurred in *P. radiata atp6* leading to alternative C-termini, but in the case of *A. macrogynus atp6,* introducing somewhat altered homolog of the new C-terminus.

Additional C-terminal coding sequence was likewise inserted into the mitochondrial intronic *rps3* gene of the Ascomycota *Ophiostoma novo-ulmi* subsp. *americana* [62]. In the latter case, the HE insertion apparently shifted a small portion of the *rps3* coding region downstream and disrupted the ORF with a premature stop codon. Accordingly, in the *P. radiata cox2* gene, the duplicated C-terminus is intervened by a 2.5 kb sequence containing catalytic HE domains, and is fused after a premature stop codon in the first C-terminus.

The group I and II type self-splicing introns and HEs are interlinked since the self-splicing introns need endonuclease activity to assist splicing of the transcribed intronic RNA [58]–[60]. If the numerous HE motifs in the *P. radiata* mtDNA are active, the homing processes may modify their target genes, and even the genome size and structure considerably - in the course of either a long or short evolutionary time period - as is seen in the mt genomes of the animal-pathogenic *Cryptococcus* spp. [53], [54]. Another function for the multiple HE domains could be regulation of transcription of their target genes, which is observed for bacterial viruses [60].

Considering the density of group I type introns (26) and their high frequency of HE domains in the core genes of *P. radiata* mtDNA, it is well established to expect that if functional, the HEs could assist in intronic RNA splicing and thereby affect transcription of their target genes, with a possibility for alternative intron splicing. The mitochondrial Complex IV cytochrome oxidase subunit 1 encoding *cox1* gene of *P. radiata* is particularly interrupted by long self-splicing introns (13) containing multiple HEs, as seemingly is general in Agaricomycota mtDNAs [24]–[28], [57]. In *P. radiata* mtDNA, *cox1* is apparently the first replicated and transcribed gene in sense (leading strand) orientation (Figure 1). Whether regulation of *P. radiata* mtDNA gene expression is mediated by the multiple introns and their identified HEs will be of our future concern.

### Plasmid-originating Features

Although mitochondrial plasmids have been sequenced, and plasmid-originating genes are identified in fungal mtDNAs [8], [23]–[28], [50], [63], [64], our sequence analyses supported no individual plasmid dsDNA features in the *P. radiata* mtDNA. We were also unable to detect integrated-plasmid like sequence regions with inverted repeat ends as has been reported for other Agaricomycota species, i.e. *A. bisporus* [23], *M. perniciosa* [24] and *A. aegerita* [64]. However, putative and degenerative plasmid and viral-originating features, such as the *cob* gene intron-located reverse transcriptase (RT, RNA-directed DNA polymerase), and DNA polymerase B encoding *dpob* genes were identified in the *P. radiata* mtDNA, thereby possibly indicating previous plasmid-transmitted DNA integration events. The mitochondrial type II intron-homing and retrovirus-related reverse transcriptase [65], [66] may function in plasmid integration to the mtDNA, and due to template-switching capacity, intron loss and gain to the mitochondrial genomes may occur.

### tRNA Assembly, Codon Usage and Phylogeny

Fungal and animal mitochondrial genomes generally have a single tRNA gene for each synonymous protein-coding codon [67]. This also applies to the mitochondria of Basidiomycota, and implies extensive codon third nucleotide (wobble) pairing, similar to that observed for the tRNAs of the bacterium *Mycoplasma capricolum* [68], i.e. the tRNA anticodon first nucleotide (anticodon wobble) U pairs with all four third position nucleotides, and the first anticodon G pairs with third codon position C or U nucleotides.

Assuming that these codon/anticodon recognition rules apply, the predicted tRNA set of *P. radiata* mtDNA is sufficient to translate its conserved mitochondrial proteome, except for the codons Ile AUA (274) and Trp UGA (1), which would require unusual A•G and A•C wobble-pairing to their respected tRNA anticodons. However, we infer from Basidiomycota tRNA-Met and tRNA-Ile multiple sequence alignments that one of the *P. radiata* mtDNA tRNAs with CAU anticodon is in fact a tRNA-Ile gene, and the predicted anticodon is likely edited. Likewise, based on the lack of UGA codons in *P. radiata* mtDNA conserved ORFs, presence of the canonical CCA anticodon in tRNA-Trp, and the high bias towards low GC-content, we infer that *P. radiata* mitochondrial genome does not utilize the Mold, Protozoan, and Coelenterate Mitochondrial Code (NCBI translation table 4) in which UGA encodes Trp.

From the frequency of UGA codons in fungal mtDNA-encoded proteomes, we infer that UGA has been assigned to Trp multiple times in the evolution of Basidiomycota mitochondrial genomes, i.e. in the lineage leading to the Pucciniomycotina genus *Phakopsora*, and in the lineage leading to the Agaricomycotina genus *Moniliopthora*, but not in the lineage leading to *Phlebia.* Likewise, we infer from sequence alignments of Basidiomycota *cox3* genes that in *C. neoformans*, UGA induces a +1 nt frameshift, which restores sequence homology for the last 16 codons of the gene in reference to other Basidiomycota *cox3* genes. We conclude that with this repertoire of mtDNA-encoded tRNAs, the mitochondria of *P. radiata* do not require tRNA import from the cytosol.

### mtDNA Proteome Phylogeny

Maximum-likelihood and Bayesian inference approaches of the Basidiomycota mtDNA-encoded proteomes resulted with well-supported and systematically consistent evolutionary trees in line with current multigene-based fungal taxonomies [69], [70], both in respect to fungal phyla (-mycota) and within subphyla (-mycotina) (Figure 5). *P. radiata* mt proteome grouped together with other Agaricomycotina species, nearest to *Ganoderma* spp. and *T. cingulata*, which also belong to the same taxonomic class (Agaricomycetes) and share similar, wood-decaying white-rot saprobic lifestyle with *P. radiata*. The opportunistic pathogen *C. neoformans* was the only exception in protein phylogeny by falling outside the subphylum Agaricomycotina, which is consistent with our mtDNA proteome ORF codon usage evolutionary analysis (Figure 4). Multi-protein Bayesian evolutionary analysis positioned the yet *insertae sedis* subphyla Kixcellomycotina (*Zancudomyces (Smittium) culisetae*) nearest to Glomeromycota, and Mucoromycotina (*Rhizopus oryzae*) and Mortierellomycotina (*Mortierella verticillata*) together between Glomeromycota and Dikarya (Figure 5). Otherwise the relationships between extant taxons were well resolved, thus indicating a strong signal for a single common origin of the Basidiomycota and fungal mt genomes.

## Conclusions

Mitochondria are numerous in eukaryotic cells and thereby, mitochondrial genomes as well have high cellular copy numbers. Our study confirms the high degree of variety of fungal mtDNAs in genome structure and size, gene order and location, and exon-intron structure of the protein-coding genes. This indicates that for mtDNA, continuous and adaptive modifications are allowed, including mobile genetic elements and signs of recombination events. Several features in the *P. radiata* mtDNA support such genetic flexibility and repair mechanisms, and regulation of transcription. Existence of the long inverted-duplicated region, frequency of repetitive sequence motifs, and especially the abundance of intron-homing endonucleases support these conclusions. Surprisingly, these features of *P. radiata* mtDNA, together with the large genome size, are shared with fungal, plant and algae mtDNAs.

Accurately characterized reference genomes including the mtDNAs are currently needed to aid in *de novo* sequencing and evolutionary studies of fungi. The novel *P. radiata* mtDNA features observed in our research indicate a general phenomenon for evolutionary pressure and genome diversity in mitochondrial genomes, not being as stable and compact integrities as previously considered. The fungal mtDNAs could thus serve as sources for evolutionary and biochemical studies of genetic mobile elements, intron loss and gain, virulence and adaptation, and targeted genetic engineering by the use of homing endonucleases.

## Supporting Information

Table S1 **Intronic and additional ORFs annotated in the** ***P. radiata*** **mtDNA.**
(XLSX)

Table S2 **ORFs continuing from coding sequence exons into putative intronic regions.**
(XLSX)

Table S3 **Root mean squared difference distance matrix of Basidiomycota conserved codons in the protein-coding ORFs.**
(XLSX)

Table S4 **Sum squared difference distance matrix of Basidiomycota conserved codons in the protein-coding ORFs.**
(XLSX)

Table S5 **Intronic homing endonuclease (HE) domains in the** ***P. radiata*** **mtDNA.**
(XLSX)
